# Essential Roles of Peroxiredoxin IV in Inflammation and Cancer

**DOI:** 10.3390/molecules27196513

**Published:** 2022-10-02

**Authors:** Pratik Thapa, Na Ding, Yanning Hao, Aziza Alshahrani, Hong Jiang, Qiou Wei

**Affiliations:** 1 Department of Toxicology and Cancer Biology, University of Kentucky College of Medicine, 1095 Veterans Dr, Lexington, KY 40508, USA; 2 Markey Cancer Center, University of Kentucky, 800 Rose Street, Lexington, KY 40536, USA

**Keywords:** peroxiredoxin, hydrogen peroxide, oxidative protein folding, inflammation, cancer

## Abstract

Peroxiredoxin IV (Prx4) is a 2-Cysteine peroxidase with ubiquitous expression in human tissues. Prx4 scavenges hydrogen peroxide and participates in oxidative protein folding in the endoplasmic reticulum. In addition, Prx4 is secreted outside the cell. Prx4 is upregulated in several cancers and is a potential therapeutic target. We have summarized historical and recent advances in the structure, function and biological roles of Prx4, focusing on inflammatory diseases and cancer. Oxidative stress is known to activate pro-inflammatory pathways. Chronic inflammation is a risk factor for cancer development. Hence, redox enzymes such as Prx4 are important players in the crosstalk between inflammation and cancer. Understanding molecular mechanisms of regulation of Prx4 expression and associated signaling pathways in normal physiological and disease conditions should reveal new therapeutic strategies. Thus, although Prx4 is a promising therapeutic target for inflammatory diseases and cancer, further research needs to be conducted to bridge the gap to clinical application.

## 1. Introduction

Reactive oxygen species (ROS) are oxygen-containing molecules that are unstable and highly reactive. Some examples of ROS include superoxide radical (O_2_^·−^), single oxygen (^1^O_2_) and hydroxyl radical (^·^OH). ROS are natural byproducts of certain cellular processes such as aerobic respiration in the mitochondria and peroxisome activity. Cells can also produce ROS in response to tissue damage or inflammation caused by external agents such as pathogens or UV radiation. Living systems use ROS for regulation of cell signaling and defense against microorganisms. However, excess ROS can damage DNA, RNA, proteins and lipids. The reduction-oxidation (redox) imbalance and/or the disruption in the regulation of cell signaling by ROS is known as oxidative stress, and it is an important risk factor for disease and ageing. Hence, it is critical to maintain a proper balance in the levels of ROS in the body.

The molecules utilized by the body to prevent or neutralize excess ROS and repair damage caused by oxidative stress are known as antioxidants. Living systems have evolved to possess several different types of enzymatic and non-enzymatic antioxidants. Some examples of antioxidants include glutathione (GSH), thioredoxin (Trx), ascorbic acid (vitamin C), superoxide dismutase (SOD), catalase, glutathione peroxidase (GPx) and Peroxiredoxin (Prx). Glutathione is the most abundant antioxidant in cells [1]. GSH can directly reduce various ROS and reactive nitrogen species (RNS), and it serves as a co-factor for several antioxidant enzymes. Trx provides electrons to a broad spectrum of proteins including antioxidant enzyme Prxs and redox-sensitive transcription factors Nrf2 and NF-κB [2,3]. SOD catalyzes the conversion of superoxide anion radicals to hydrogen peroxide (H_2_O_2_) and molecular oxygen [4]. Catalase reduces hydrogen peroxide to water and molecular oxygen [5]. GPx reduces hydrogen peroxide to water and lipid peroxides to their corresponding alcohols [6]. Prx, first identified in 1987 [7], catalyzes the reduction of hydrogen peroxide, alkyl hydroperoxide and peroxynitrite [8,9,10]. Prxs contain Cys-SH on their catalytic site. To date, six Prxs have been identified in mammalian cells. They are grouped as typical 2-Cys Prxs (Prx1–4), atypical 2-cys Prxs (Prx5) and 1-Cys Prx (Prx6) [11]. All Prx isoforms contain a conserved cysteine residue, called peroxidatic cysteine (Cp), on the N-terminus. Prx1–5 also contain a second conserved residue, resolving cysteine (Cr), on the C-terminus. Prx1–4 form homodimers upon oxidation of Cp via disulfide bonds with the Cr of another unit [11]. Prx5 is considered atypical because it has low sequence homology with Prx1–4, and it forms an intramolecular disulfide bond upon oxidation [12]. Since Prx6 contains only one catalytic Cys, oxidized Prx6 forms a heterodimeric disulfide linkage with thiol groups from other molecules [13]. Prx4 is unique in the Prx family as it is the only isoform that is found in the endoplasmic reticulum (ER). Prx4 may also be secreted into the extracellular matrix. In this review, we will discuss the biochemical and structural properties of human Prx4, its interaction with other proteins, and its role in various diseases.

## 2. Prx4 Structure and Function

### 2.1. Structure

**Figure 1:**
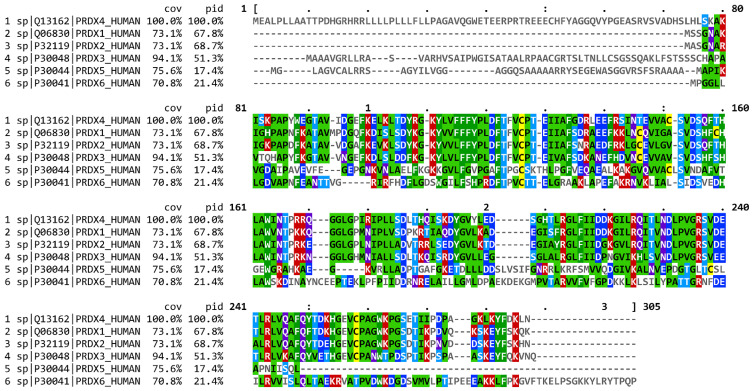
Multiple sequence alignment of human Prxs colored by consensus generated using MView. Coverage and percentage identity values are indicated by cov and pid, respectively. Cysteines are highlighted with yellow color. For other residues, Red = positively charged, blue = negatively charged, purple = polar, and green=hydrophobic.

**Figure 2:**
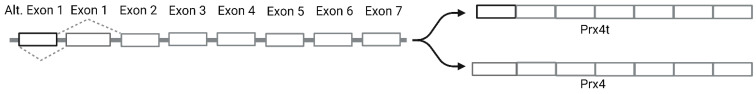
Schematic representation of alternative splicing of Prx4. Systemic Prx4 contains exon 1, whereas Prx4t expressed in mature testes contains alternative exon 1.

Comparison of mammalian Prx4 protein sequence shows that human Prx4 has a highly similar amino acid sequence as commonly used animal models such as mice, rat and zebrafish (Table 1) [14]. In addition, Prx4 has approximately 68% homology with human Prx1 and Prx2, and 52% homology with Prx3 (Figure 1) [15,16,17]. Prx4 is found on the X chromosome (Xp22.11) and the longest transcript contains seven exons. Somatic Prx4 contains conventional exon 1 and exons 2–7 [18]. However, alternative splicing of Prx4 can occur in the testes (Figure 2). Prx4t in sexually mature testes contains an alternative exon 1 along with exons 2–7 [19].

Prx4 is localized mainly in the endoplasmic reticulum [20,21]. It is also secreted into the extracellular matrix [20,22,23]. The unique extended N-terminal region in Prx4 allows for translocation of Prx4 across the ER membrane into the luminal space [21]. Since Prx4t lacks the N-terminal signal peptide, it is found in the cytosol. ER localization of Prx4 despite lacking the canonical ER retention ‘KDEL’ signal is due its interaction with PDI and ERp44. In HeLa cells, secretion due to overexpression of Prx4 could be suppressed by overexpression of ER proteins ERp44 and PDI [24]. Knockdown of ERGIC-53 in HeLa cells or treatment with 4-phenylbutyrate induces secretion of Prx4, further confirming the importance of ERp44 in its ER localization [25]. 

Human Prx4 has been crystallized in both oxidized and reduced states [26,27]. In both states, Prx4 was crystallized as a decamer, composed of five dimers. Similar to other Prxs, each subunit contains the thioredoxin fold (ββαβαβαβαββαα). A dimer acts as a catalytic subunit. In the dimer, β1- and β8- strands of partner subunits interact with each other whereas a central twisted β-sheet is surrounded by α-helices. Cp is located in α2 helix in a pocket surrounded by β4- and β5- strands and α3- and α5- helices. Cr is located in a flexible loop between α5- and α6- helices. In the reduced state, there is a distance of 13 Å between the Cp of one subunit and Cr of another. Upon oxidation, there is local unfolding in α2- helix, facilitating repositioning of Cp for the formation of disulfide bond with Cr of partner subunit. Cao et al. suggest that the higher stability of Prx4 decamer compared to other Prxs could be attributed to Phe-122 displacing Pro-260 to maintain the hydrophobic interaction between subunits [27]. In addition, compared to other 2-Cys Prxs, Prx4 has a unique N-terminal sequence that is approximately 40 aa long. Wang et al. found that deletion of these N-terminal residues resulted in decreased stability of Prx4 decamers upon oxidation by H_2_O_2_ [26].

### 2.2. Biochemical Function

**Figure 3:**
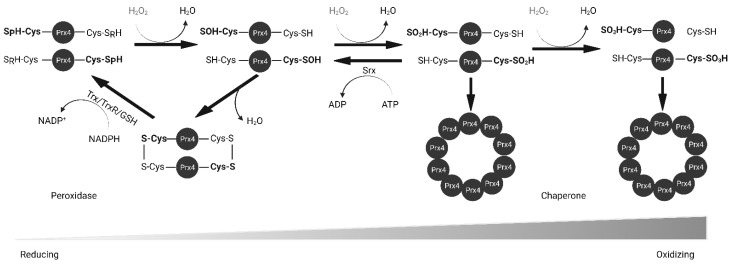
Reduction of H_2_O_2_ by Prx4. Peroxidatic cysteine (Cys-SpH) is oxidized to sulfenic acid and either resolved and recycled with the help of Trx or GSH, or further oxidized into sulfinic and sulfonic acid forms. Srx reduces sulfinic acid. Prx4 loses peroxidase activity and gains chaperone activity with increasing oxidizing environment. Trx, thioredoxin; TrxR, thioredoxin reductase; GSH, glutathione; Srx, sulfiredoxin.

Prxs are thiol-based peroxidases and cysteine residues that are utilized for redox purposes. Prx1–4 have two cysteine residues that participate in catalysis: peroxidatic Cys (Cp) and resolving Cys (Cr). The N-terminal peroxidatic cysteine has a pKa of 5–6, much lower than the normal Cys pKa of 8–9 because it is stabilized by neighboring arginine and threonine residues [28]. The lower pKa makes it more reactive to H_2_O_2_. As shown in Figure 3, upon contact with peroxide, the thiol group of Cp in Prx4 is oxidized to sulfenic acid (Cys-SOH). The oxidized Prx4 forms a disulfide bond with Cr of another polypeptide resulting in a stable homodimer whereas H_2_O_2_ is reduced to water. This disulfide can be reduced by Trx, GSH, PDI or other ER oxidoreductases [22,23,29]. However, at higher concentrations of H_2_O_2_, Prx4 is further oxidized into sulfinic acid (Cys-SOOH) and sulfonic acid (Cys-SOOOH) leading to loss of enzyme activity [30]. Sulfinic acids can be reduced by Sulfiredoxin (Srx) in an ATP-dependent manner [31,32]. Unlike other 2-Cys Prxs, over-oxidized Prx4 can maintain stable decamers through hydrophobic interactions between subunits and disulfide bond between non-catalytic N-terminal cysteine residue [26,27]. Later studies revealed that mutation of Cp and Cr alone or in combination prevents the formation of decamers [33]. The rate constant for H_2_O_2_ reduction of Prx4 is 2.2 × 10^7^ M^–1^ S^–1^, which is comparable to that of catalase and several orders of magnitude higher than that for reduction by GSH (0.87 M^–1^ S^–1^) or Trx (1.05 M^–1^ S^–1^) [26,34,35,36]. This rate is also significantly higher than that of another ER antioxidant, GPx8 (95 M^–1^ S^–1^) [37].

**Figure 4:**
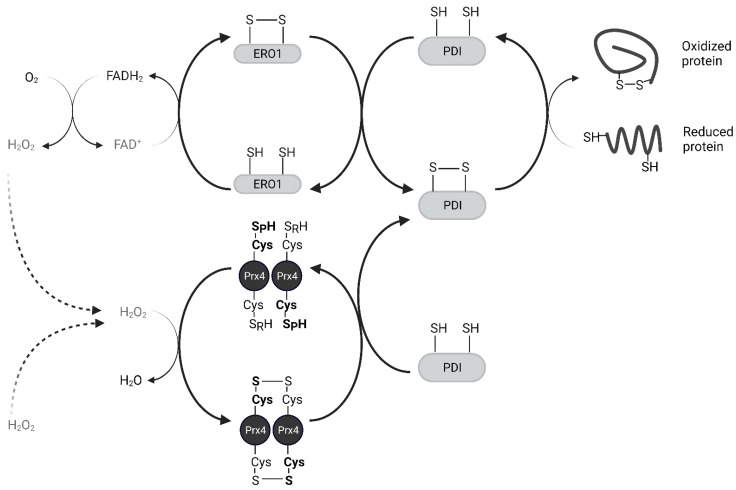
Prx4 mediates nascent peptide folding in the endoplasmic reticulum. Prx4 neutralizes H_2_O_2_ including those produced by Ero1. Oxidized Prx4 transfers disulfides to protein disulfide isomerases which catalyze the formation of disulfide bonds in nascent proteins. Only the catalytic dimer of Prx4 is shown for simplicity. Ero1, ER oxidoreductin 1; PDI, protein disulfide isomerase.

Oxidized Protein Disulfide Isomerase (PDI) family proteins introduce disulfide bonds in nascent proteins in the ER [38]. ER oxidoreductin 1 (Ero1) re-oxidizes PDI using its cofactor Flavin Adenine Dinucleotide (FAD) and releases H_2_O_2_ as a byproduct [39]. For every disulfide bond introduced into nascent proteins, one molecule of H_2_O_2_ is produced [40]. Prx4 scavenges these H_2_O_2_ molecules and prevents their accumulation. Prx4 also contributes to the folding of plasma membrane proteins and secreted proteins, acting upstream of PDIs. The interaction of Prx4 with PDIs and other ER-associated proteins increases with its oxidation, likely through its recognition of Trx-domain within PDIs [33,41]. Oxidized Prx4 engages in thiol-disulfide exchange with reduced PDIs, which results in restoration of activity for both (Figure 4) [29]. Loss of an Ero1 gene or Prx4 alone has no apparent phenotype in mice, but loss of both interferes with collagen synthesis and compromises the extracellular matrix [42]. Thus, Prx4 neutralizes peroxides in its reduced state and promotes protein folding in its oxidized state. H_2_O_2_ and Srx act as an on-off switch in regulating these activities [43].

The mechanism of Prx4 secretion and the function of secreted Prx4 are not well understood. Okado-Matsumoto et al. have previously shown that Prx4 can be secreted in both reduced and oxidized forms [23]. Since the reduced form of extracellular Prx4 binds to heparin and human umbilical vein endothelial cells in a manner similar to another extracellular antioxidant SOD3, the authors suggest that extracellular Prx4 also protects tissues against antioxidant injury. Additionally, acute exercise is known to affect redox balance in skeletal muscle [44,45] whereas muscle cells and immune cells have been shown to secrete redox proteins such as Trx and Glutaredoxin in response to increased H_2_O_2_ [46,47]. Wadley et al. reported a significant increase in plasma Prx4 levels after high intensity exercise [48]. These studies suggest a role for Prx4 in scavenging H_2_O_2_ in the extracellular space. Furthermore, as described in more detail below, secreted Prx4 also likely plays a role in regulating inflammation via NF-κB signaling.

## 3. Prx4 and Inflammation

### 3.1. Roles of Prx4 in Inflammatory Diseases

#### 3.1.1. Diabetes and Nonalcoholic Fatty Liver Disease (NAFLD)

Yamada et al. have generated human Prx4 transgenic mice and summarized the protective role of Prx4 in various chronic inflammatory diseases [49]. In brief, they found that overexpression of hPrx4 protected the mice against streptozotocin-induced type 1 diabetes mellitus (DM), progression of hypercholesterolemia-induced atherosclerosis, high fructose diet and streptozotocin- induced Type 2 DM and NAFLD as well as methionine- and choline-deficient high-fat diet- induced NAFLD by suppressing oxidative damage, inflammation and apoptosis. However, two independent studies have described higher levels of serum Prx4 in patients with prediabetes and type 2 DM than in healthy volunteers [50,51]. It is not yet clear what the function of elevated serum Prx4 levels might be in relation to these diseases. In another study, stable knockdown of Prx4 in MIN6 β-cells increased the susceptibility of proinsulin misfolding, especially after oxidative stress [52]. Conversely, transfection of Prx4 plasmid promoted insulin folding. The authors also report that human islets from patients with Type 2 DM contain a higher fraction of overoxidized Prx4 (Prx4-SO_3_) than samples from normal individuals. Together, these studies indicate a possible role for Prx4 in regulation of glucose. 

Knockout of Prx4 or SOD1 alone did not contribute significantly to the development of liver failure in mice [53]. However, the combined silencing of Prx4 and superoxide dismutase 1 (SOD1) significantly exacerbated the effects. There was an increase in hyperoxidation of Prx 1–3, and an upregulation of ER chaperone Grp78 and ER stress response protein CHOP in the whole liver lysates of double-knockout mice, indicating the importance of Prx4 in protection against oxidative stress and ER stress. In addition, Prx4 has been suggested to play a role in suppressing ferroptosis [54]. Ferroptosis is a form of programmed cell death dependent on iron and characterized by redox imbalance and accumulation of intracellular lipid peroxides. Celastrol, a natural product isolated from medicinal plant *Tripterygium wilfordii* Hook F, protected mice against carbon tetrachloride (CCl_4_)-induced hepatic fibrosis [54]. Mechanistic studies revealed that Celastrol suppresses the antioxidant activity of Prx1, Prx2, Prx4 and Prx6 and induces accumulation of ROS and ferroptosis markers Fe^2+/^Fe^3+^ and LPO in the LX-2 cell line. This study suggests that targeting Prx4 could be a novel strategy for regulating ferroptosis. 

#### 3.1.2. Cardiovascular Diseases

Loss of Prx4 is associated with cardiac stress in the absence of QSOX1 [55]. The authors of this study found that QSOX1 participates in early protective response to acute cardiac stress. Under normal conditions, Prx4 and Ero-1α were found to be upregulated in the hearts of QSOX1 knock out mice. Induction of acute stress by intraperitoneal injections of isoproterenol resulted in a decrease of Prx4 levels and an increase in inflammation (increased galectin-3, CD68+ cells) and oxidative stress within three days of injection [55]. Prx4 also has a more direct relationship with galectin-3, which downregulates Prx4 in human cardiac fibroblasts [56]. Galectin-3 is induced in tissue injury such as heart failure, and it is known to promote fibrosis and inflammation. Human cardiac fibroblasts stimulated with galectin-3 had decreased Prx4 protein levels [56]. The study showed that knockdown of galectin-3 in vitro with siRNA for 24 h resulted in upregulation of Prx4 protein. Similarly, Prx4 was upregulated in hearts of galectin-3 knockout mice compared to wild-type (WT) mice. In spontaneously hypertensive rats, which present enhanced cardiac galectin-3, its pharmacological inhibition increased cardiac Prx4 levels and decreased oxidative stress. No association was detected between serum galectin-3 levels and serum Prx4 levels in samples from aortic stenosis (AS) patients. In an immunohistochemical analysis of myocardial biopsies of AS patients, Prx4 expression was lower in AS patients compared to control. In addition, an inverse correlation was detected between Prx4 mRNA and galectin-3 protein levels [56]. A later study found that cardiotoxicity caused by the chemotherapeutic drug doxorubicin could be reduced by upregulating Prx4 through galectin-3 inhibition [57]. Thus, Prx4 has a protective role in heart tissue.

#### 3.1.3. Cerebral Ischemia and Alzheimer’s Disease

Ischemic stroke is a common cause of death and disability. Rowe et al. discovered that human umbilical cord blood cell (HUCBC) treatment of oligodendrocytes in a non-contact co-culture model protected the cells from oxygen glucose deprivation (OGD) [58]. Subsequent microarray analysis revealed HUCBC treatment following OGD had induced Prx4 and another antioxidant, metallothionein 3, in the oligodendrocytes. The group also found that co-treatment of oligodendrocytes with inhibitor of Akt during OGD suppressed the increase in Prx4. The protective effect of HUCBC following OGD could be reversed by an Akt inhibitor or Prx4-neutralizing antibody. Similarly, another study found that Prxs are highly expressed in mesenchymal stromal cells (MSCs) [59]. The authors of this study reported MSC cells with stable expression of CCR2 to be promising for promoting neurological recovery after acute ischemic stroke. They propose that Prx4 secreted by MSCs had an important role in preserving the blood–brain barrier in this process. Silencing Prx4 in MSC-CCR2 cells increased their total ROS levels (measured using cellROX), disrupted tight junction and decreased the length of blood brain barrier marker Glut1 in brain slices, effectively reversing the effect of CCR2 expression. Thus, extracellular Prx4 has a protective role in ischemia, and it should provide an additional strategy for improving cell therapy.

Prx4 expression was detected in ependymal layer, choroid plexus, astrocytes and neurons, but it was not detected in microglia and oligodendrocytes in the normal adult mouse brain [60]. In an analysis of the postmortem brains of Alzheimer’s disease (AD) patients, Prx4 protein levels were found to be decreased [61]. The authors suggest the high oxidative stress as a result of this downregulation could potentially lead to concurrent phosphorylation of AMPK and mTOR in AD [61]. Prx4 was found to have a protective effect against amyloid beta oligomer (AβO)- and glutamate- mediated stress in vitro [62]. The treatment of mouse hippocampal neuronal HT-22 cells with 5 μM of AβO upregulated Prx4 expression in a time-dependent manner [62]. Pretreatment of cells with tauroursodeoxycholic acid (TUDCA) or N-acetyl cysteine (NAC) disrupted this increase. The study also noted that HT-22 cells stably overexpressing Prx4 had lower ROS levels and lower ER stress compared to cells targeted with siRNAs against Prx4. Prx4 overexpression also decreased AβO-induced intracellular Ca^2+^ uptake and protected against apoptotic cell death. In a different study, overexpression of Prx4 in HT-22 cells reduced glutamate-induced apoptosis by inhibiting ROS formation (measured using CM-H2DCF-DA staining), Ca^2+^ influx and ER stress [63]. β-amyloid, known to contribute to neuron degeneration, is derived from the amyloid precursor protein (APP). Triple knockdown of APP family genes APP, APLP1 and APLP2 in HEK293T cells resulted in a significant downregulation of Prx4 protein (but not mRNA) levels [64]. Together these studies suggest Prx4 plays a beneficial role against AD.

#### 3.1.4. Colitis

Two-dimensional gel electrophoresis and mass spectrometric analyses of colon tissue samples showed that Prx4 was upregulated (whereas Prx3 and Prx6 were downregulated) in ulcerative colitis patients compared to healthy controls [65]. However, this pro-inflammatory association of Prx4 contrasts with other data reporting an anti-inflammatory role. Takagi et al. report a high expression of Prx4 in the epithelial cells of the colon [66]. The treatment of mice with 2.5% dextran sulfate sodium (DSS) in drinking water for seven days resulted in significantly shorter colon length in Prx4 knockout (KO) mice than WT mice. Prx4 KO mice had greater epithelial damage and higher infiltration of neutrophils as well as elevated expression of inflammatory cytokines including TNF-α and IFN-γ. After DSS treatment, the Prx4 KO mice had a greater increased epithelial permeability than WT mice, expanded ER, increase expression of CHOP and elevated cleaved caspase 3. Without DSS treatment, there was no significant difference in collagen IV between WT and KO mice. After treatment, collagen IV increased in both groups, but compared to WT, it increased less in KO. These KO mice had fewer collagen fibers in the intercellular space than WT. Finally, Prx4 KO mice had greater induction of fibrosis-related proteins α-SMA and TGF-β than WT mice after DSS treatment. Thus, Prx4 protected mice against DSS-induced inflammation in this study [66]. Interestingly, another Prx family member, Prx1, negatively correlates with inflammation in colitis. In ulcerative colitis patient samples, increasing colitis severity was associated with increasing Prx1 expression in epithelial cells as well as in infiltrating stromal cells [67].

#### 3.1.5. Rheumatism and Other Inflammatory Conditions

A protective role for Prx4 has been suggested in osteoarthritis (OA). Overexpression of Prx4 in rat primary chondrocytes in an OA model decreased IL-1β-induced ROS production (measured using DCF-DA staining and DHE staining) and apoptosis factors that are known to contribute to cartilage degeneration [68]. This could also be partially reversed by treatment with Akt inhibitor AZD5363, suggesting Prx4 utilizes the PI3K/Akt pathway. Interestingly, two-dimensional gel electrophoresis and mass spectrometric analyses of synovial tissue samples showed that Prx4 was upregulated in rheumatoid arthritis (RA) patients compared to OA and ankylosing spondylitis (AS) [69]. Using an enzyme-linked immunosorbent assay (ELISA), the authors also detected higher Prx4 in plasma from early-stage RA patients compared to healthy controls. The function of Prx4 in the initiation of RA is not understood. The upregulation of Prx4 in RA synovial tissue was confirmed by a later study [70]. The authors also found that knockdown of Prx4 in primary fibroblast-like synoviocytes, an important cell type in synovial tissue, decreased phosphorylated PI3K and Akt and suppressed cell proliferation, migration and invasion in vitro. These effects could be reverted by Akt inhibitor MK-2206. Thus, Prx4 is a potential therapeutic target in arthritis.

It has been reported that expression of Prx4 protein is lower in alveolar macrophages of patients with the development of silicosis [71]. This suggests a role for Prx4 in suppression of inflammation in the lungs. The authors also suggest monitoring the markers of oxidative stress as prognostic and predictive factors for silicosis.

Prx4 expression is increased in mRNA and protein levels during LPS-induced differentiation of B cells into plasma cells [72]. However, Prx4 is not essential for this differentiation, as Prx4 knockout cells also differentiated normally.

In a wound healing model, overexpression of Prx4 promoted skin wound healing in adult and aged mice by reducing oxidative stress and neutrophils numbers and by increasing macrophage infiltration and growth factor levels [73].

### 3.2. Signaling Pathways Regulated by Prx4 in Inflammation

#### 3.2.1. NF-κB

Transcription factor NF-κB is a key regulator of inflammation that has been implicated in the initiation of various cancers [74,75,76]. In general, intracellular Prx4 appears to suppress NF-κB whereas extracellular Prx4 activates NF-κB.

Jin et al. discovered that cytosolic Prx4, which they called AOE372, negatively regulated NF-κB activation [77]. Overexpression of Prx4 in HeLa cells significantly suppressed HIV-1 Tat-, Tumor necrosis factor (TNF)- and 12-*O*-tetradecanoylphorbol-13-acetate (TPA)-dependent activation of NF-κB. NF-κB-dependent reporter assays showed that Prx1 and Prx4 inhibited NF-κB synergistically. The authors suggested that Prx4 could affect phosphorylation of IκB-α as a potential mechanism. 

In HEK293 cells with stable expression of immune receptor NOD2, treatment with microbial product muramyl dipeptide (MDP) upregulated Prx4 expression [78]. Interestingly, the authors also found that the loss of Prx4 in HEK293 cells enhanced NF-κB activation upon treatment with MDP, suggesting Prx4 negatively regulates NF-κB signaling.

In the large yellow croaker, *Pseudosciaena crocea*, bacterial infection upregulated Prx4 in the spleen [79]. When the fish were injected with Prx4-siRNA before the bacterial challenge, NF-κB binding activity in the spleen increased and mRNA levels of TNF-α and CC chemokine increased, whereas IL-10 decreased. Opposite results were seen in fish with injected with recombinant Prx4. Thus, the result indicates that in *P. crocea*, Prx4 negatively regulates the activity of NF-κB, downregulates pro-inflammatory cytokines and upregulates anti-inflammatory cytokines in response to bacterial infection. The negative regulation of NF-κB by Prx4 in *P. Crocea* was later confirmed in another study [80].

In the *Drosophila* model, flies overexpressing Prx4 at high levels (over 5-fold) exhibited upregulation in the mRNA expression of AttD, Dipt and Mtk (some of the targets of *Drosophila* NF-κB), as well as upregulation of TotA, which is a downstream target of the JAK/STAT pathway [81]. However, injection of Prx4 into the body cavity of *Drosophila* did not yield significant changes in mRNA levels of AttD, Dipt and Mtk (it did upregulate TotA). Hence, the authors suggest that only intracellular Prx4 is involved in the activation of immune response.

In contrast, Haridas and colleagues reported that secreted Prx4 activated NF-κB in human cell lines [22]. They confirmed the secretion of Prx4 from Jurkat and HL-60 cells into conditioned medium. Treatment of human myeloid cells U-937 with Prx4 for 30 min showed Prx4 activated NF-κB in a dose-dependent and time-dependent manner. NF-κB activation peaked at 4 h post-treatment which coincided with the complete degradation of IκB-α. Prx4 treatment also increased NF-κB dependent luciferase activity by seven-fold. Zhao et al. studied the pro-inflammatory properties of peroxiredoxins in mouse macrophages in vitro [82]. They found that 24 h treatment of RAW264.7 cells with different concentrations of recombinant mouse Prx4 ranging from 1 nM to 50 nM had no effect on cell viability. However, at the higher concentrations of 20 nM and 50 nM, Prx4 induced a significant increase in NO levels in the conditioned medium in a dose-dependent manner. Finally, a 24 h treatment with 20 nM Prx4 significantly increased TLR4 expression and the nuclear translocation of NF-κB p65 subunit. These data suggest that extracellular Prx4 could have a pro-inflammatory effect through TLR4/ NF-κB signaling activation. 

#### 3.2.2. Inflammasome

Inflammasomes are protein complexes assembled by the innate immune system to regulate inflammatory response. Inflammasomes trigger maturation of proinflammatory cytokines IL-1β and IL-18 through activation of caspase-1 [83]. Prxs have been increasingly associated with regulation of inflammasomes. Activation of inflammasomes NLRP3, NLRC4 or AIM2 in murine macrophages in vitro caused secretion of Prx1, Prx2, Prx5 and Prx6 [84]. Downregulation of Prx1 was suggested to transcriptionally inhibit NLRP3 inflammasome expression in intestinal inflammation [85]. Serum Prx1 was found to promote inflammation in acute liver injury through NLRP3 inflammasome signaling [86]. Knockdown of Prx3 in the liver aggravated acetaminophen-induced liver injury and this was associated with increased markers of NLRP3 inflammasome activation [87]. A curcumin analogue, AI-44, prevented activation of procaspase 1 by promoting its interaction with Prx1 [88]. Similarly, a loss of phospholipase A2 activity in Prx6 in primary endothelial cells protected against LPS-induced upregulation of NLRP3 [89].

Lipinski et al. report that Prx4 limits caspase-1 activation and restricts inflammasome-mediated signaling by extracellular vesicles [90]. The authors found that when challenged with sub-lethal dose of LPS intraperitoneally, Prx4-null mice had increased weight loss, increased serum TNF-α, IL-1β, CXCL1 and delayed restoration of weight than WT mice. This could be prevented by treatment with an interlukin-1-receptor antagonist. In mice lacking Prx4 in myeloid cells, the results found in whole body knockout was replicated suggesting a crucial role of myeloid cells in Prx4-mediated protection. In vitro, oxidized Prx4 decamer complex directly inhibited caspase-1 activity through interacting with redox sensitive C397 of caspase-1. Their studies also show inflammasome-activated cells secreting Prx4 in extracellular vesicles along with components of inflammasome. Presence of Prx4 in extracellular vesicles caused a lower pro-inflammatory response in recipient cells and mice. Thus, Prx4 negatively regulates caspase-1 and IL-1β activation to lower the inflammatory response. 

#### 3.2.3. Others

Cyclooxygenase-2 (COX-2) catalyzes a critical step in the synthesis of prostaglandins and other prostanoids, and it is a target of non-steroidal anti-inflammatory drugs (NSAIDs) [91]. In a hyperosmotic medium, which was used as an in vitro model for studying dry eye disease, the expression of COX-2 was upregulated, whereas Prx4 and SOD1 were downregulated in human corneal epithelial cells [92,93]. However, treatment with the small molecule L-carnitine, or with pterostilbene, a natural component of blueberries, restored the expression of these enzymes back to the levels seen in isosmotic condition [92,93]. Thus, suppression of inflammation combined with maintenance of Prx4 and other antioxidants seems vital to the treatment of dry-eye disease.

Although no causal relationship has been reported between Prx4 and interleukin 6 (IL-6), there appears to be a negative correlation. TNF-α, IL-1β and IL-6 are all upregulated in human corneal epithelial cells under hyperosmotic conditions. Treatment with pterostilbene reduces their expression whereas Prx4 is upregulated [92]. Similarly, in the goldfish animal model, exposure of the fish to 10 ng/L of the organotin triphenyltin (TPT) significantly lowered the mRNA levels of antioxidants including Prx4 while increasing the secretion of TNF-α, IL-1β and IL-6 in the serum [94]. Finally, in both acute and chronic treatments with DSS, pro-inflammatory cytokines TNF-α, IFN-γ and IL-6 were downregulated at mRNA level whereas Prx4 mRNA and protein were significantly upregulated in the colons of Prx6 KO mice compared to WT mice [95]. Thus, intracellular Prx4 has a negative correlation with IL-6. However, extracellular Prx4 activates IL-6. Recombinant mouse Prx4 induced secretion of TNF-α and IL-6 upon addition to murine macrophages RAW264.7 [82].

In kuruma shrimp (*M. japonicus*), bacterial infection upregulated shrimp Prx4 at transcript and protein levels [96]. Knockdown of Prx4 with dsRNA injection increased the bacterial number in shrimp and decreased overall survival. Mechanistic studies showed that nuclear translocation and phosphorylation of STAT increased upon infection in the control group but was suppressed in the Prx4-depleted group. STAT-activation could also be blocked by injection of a Prx4 antibody prior to bacterial challenge, suggesting extracellular Prx4 was responsible for STAT activation. Similarly, treatment with purified shrimp Prx4 activated STAT whereas mutant Prx4 modified on both catalytic cysteines failed to do so. The authors also report that extracellular Prx4 acted through the receptor Domeless to activate JAK/STAT as knockdown of this receptor, which blocked nuclear translocation of STAT [96]. Thus, Prx4 contributes to the antibacterial immunity of shrimp through the JAK/STAT pathway.

## 4. Prx4 and Cancer

**Figure 5:**
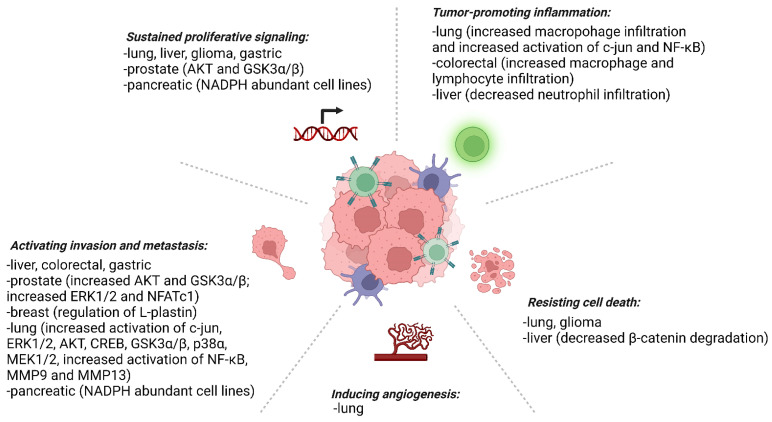
Prx4 promotes the hallmarks of cancer in different cancer types. Known aberrant signaling pathways and markers are shown in parentheses.

Prx4 has been found to be upregulated in the majority of cancers. Below, we briefly discuss roles of Prx4 in major cancer types and their tumor microenvironment (Figure 5).

### 4.1. Prostate Cancer

Prx4 is pro-tumorigenic in prostate cancer. Studies have reported that there is an upregulation of Prx4 in human prostate cancers [97,98]. Prx4 upregulation enhances proliferation of prostate cancer cell lines DU145 and LNCaP in vitro [97]. Incidentally, they also discovered Prx4 overexpression to correlate negatively with TMPRSS2-ERG gene fusion, a highly common genomic alteration present in prostate cancer patients. In a different study, Prx4 overexpression in prostate cancer was associated with increase in tumor stage, increase in Gleason sum score and increase in age at prostatectomy [98]. Knockdown of Prx4 in prostate cancer cell line PC3 to reduce Prx4 secretion repressed the ability of cancer cells to induce osteoclastogenesis in vitro and osteolysis in vivo [99]. The authors have suggested that Prx4 deficiency might interfere with the ERK1/2 signaling pathway and NFATc1 nuclear translocation. In addition, Prx4 has been found to be upregulated in prostate cancer and other cancers that commonly metastasize to bone [100]. One possible mechanism for Prx4 upregulation is through androgen receptor signaling. Treatment of the LNCaP cell line with synthetic androgen R1881 led to a dose-dependent increase in Prx4 expression [101]. Depletion of Prx4 in LNCaP and DU145 cell lines decreased cell proliferation, migration and invasion, likely through decreased activation of Akt and GSK3 signaling pathways. Prx4 depletion also sensitized the cancer cells to radiation in vitro and in mouse xenograft model [101]. Other Prxs, such as Prx1 also plays a role in prostate cancer development. Prx1 induces HIF1α and VEGF expression in prostate cancer cell lines [102]. Knockdown of Prx1 in prostate cancer cell lines and subcutaneous injection into mice resulted in a reduced tumor vasculature formation [103]. Further experiments revealed that Prx1 stimulated endothelial cell migration and differentiation through Toll Like Receptor 4 (TLR-4) signaling.

### 4.2. Breast Cancer

Prx4 is overexpressed in breast cancer samples compared to normal tissues [104,105]. Immunohistochemical staining revealed that Prx4 is overexpressed in triple negative breast cancer (TNBC) cases compared to the non-TNBC cases [106]. The TNBC group had lower oxidative stress, as measured by 8-Hydroxydeoxyguanosine (8-OHdG) staining, and poor breast-cancer specific survival. Another study confirmed using the UALCAN database that Prx4 expression at the transcript level was highest in the TNBC group, followed by Her-2 positive group [107]. This upregulation of Prx4 was positively correlated with shorter disease-free survival and poor overall survival. Similar to prostate cancer, breast cancer cells also utilize secreted Prx4 to mediate osteoclastogenesis. Knockdown of Prx4 in MDA-MB-231 cells significantly reduced osteoclast formation in vitro [99]. In addition, their bioinformatics analysis revealed that patients with lower Prx4 expression in primary tumor were less likely to develop metastasis at five years compared to those with higher Prx4 expression. Tiedemann et al. have suggested that Prx4 influences L-plastin expression, with both proteins involved in mediating breast cancer-induced osteolysis in vivo [100]. When MDA-MB-231 cells lacking both L-plastin and Prx4 were injected into CD-1 immunodeficient mice, a complete loss of osteolysis was observed. Prx4 also contains single-nucleotide polymorphisms (SNPs) associated with clearance of docetaxel [108]. Finally, exposure of cell line MDA-MB-231 to increasing concentrations of docetaxel followed by whole exome sequencing at five different stages revealed that there was a copy number loss of a number of genes on the X chromosome, including Prx4 at stage 2/3 [109]. Thus, Prx4 is a novel therapeutic target for the treatment of breast cancer.

Other Prxs are also implicated in breast cancer development. Degradation of Prx1 is associated with pro-tumorigenic activation of macrophages [110]. In mammary fibroblasts, loss of Prx1 also promoted the transition into cancer-associated fibroblast-phenotype under oxidative stress through activation of c-JUN kinases [111]. In a DMBA-induced breast cancer model, zinc consumption through diet-reduced Prx1 expression in the mammary tissue, resulted in higher oxidative stress and the loss of the protective effect of lactation in cancer development [112]. Finally, Prx2 was found to be upregulated in tumor interstitial fluids compared to normal interstitial fluids in breast cancer patients, suggesting the potential of Prx2 as a diagnostic marker for breast cancer [113].

### 4.3. Lung Cancer

In general, Prx4 has been found to be pro-tumorigenic in lung cancer. Prx4 is the primary substrate of Srx in lung cancer cells [114]. Knockdown of Srx or Prx4 represses anchorage independent colony formation and cell invasion of A549 and H226 cells [114,115]. Accordingly, disruption of Srx-Prx4 axis reduces tumor growth and metastasis in vivo in mouse models. Furthermore, knockdown of Prx4 affects a large number of kinase signaling pathways including c-Jun, ERK1/2, Akt, CREB, GSK3α/β, p38α and MEK1/2, among others.

Hwang et al. reported that strong Prx4 expression in stage II non-small cell lung cancer (NSCLC) patients correlated with short disease-free survival in the squamous cell carcinoma (SCC) subgroup but not in the adenocarcinoma (LUAD) subgroup [116]. A urethane (1 g/kg) i.p. injection of non-transgenic control and human Prx4-expressing transgenic mice for 16 weeks resulted in significantly more and bigger tumors in the transgenic group [117]. This increase in tumor burden was attributed to the suppressed apoptosis and enhanced proliferation in the tumors in the transgenic group. The authors also found that the hPrx4 expressing group had higher microvascular permeability, macrophage infiltration, MMP9, MMP13 and IL-1β production and significantly lower oxidative stress as indicated by lower 8-OHdG staining and serum malondialdehyde concentration. In NSCLC cell lines A549 and H460, irradiation upregulated TRIAP1 and several antioxidants including Prx4 [118]. Knockdown of TRIAP1 sensitized these cells to radiation as the induction of Prx4 and other antioxidants was disrupted. Thus, Prx4 promotes chemically induced tumorigenesis and radioresistance in NSCLC.

However, an anti-tumorigenic role of Prx4 in lung cancer has been found in LUAD. Shioya et al. reported that weak Prx4 expression correlated positively with poor differentiation and high invasiveness of tumors in stage I LUAD [119]. A later study of stage I LUAD found that weak Prx4 expression correlated positively with WT status of EGFR [120]. The authors have suggested that the combination of weak expression of Prx4 with high MIB-1 labelling index and/or WT status of EGFR may be a useful tool to predict poor disease-free survival in early-stage LUAD [119,120].

Prx1 and Prx5 are also pro-tumorigenic in lung cancer. Prx1 has been reported to be induced by hypoxia in lung cancer cells in vitro [121,122]. Loss of Prx5 has been shown to promote M2 polarization of macrophages and lung cancer development [123]. M2-like polarization could be inhibited by treatment of the cells with antioxidant NAC to suppress ROS (measured using CM-H2DCF-DA staining). The authors of this study suggest a new potential therapeutic approach of altering macrophage polarization through ROS levels.

### 4.4. Colorectal Cancer

Prx4 has an oncogenic role in colorectal cancer (CRC). The analysis of CRC patient specimens showed that Prx4 mRNA and protein expression were significantly higher in CRC samples compared to adjacent normal tissue [124]. The study also found a significant positive correlation between Prx4 protein expression in CRC tissues and the depth of invasion, lymph node metastasis, tumor stage and shorter survival; however, univariate and multivariate analyses revealed that Prx4 was not an independent unfavorable prognostic factor for the survival of CRC patients. Li et al. found through hierarchical cluster analysis using data from cDNA microarray and subsequent quantitative PCR that Prx4 was expressed at a significantly higher levels in primary CRC tumors with liver metastasis than in tumors without metastasis [125]. Prx4 was one of 18 proteins differentially expressed between tissue samples of stage I and stage IV colorectal cancer [126]. Prx4 upregulation in advanced stages of CRC was identified using two-dimensional gel electrophoresis and mass spectrometry and validated in tissue microarrays. Knockdown of Prx4 in DLD-1 cells induced G1/S arrest and reduced migration and invasion in vitro. Western blot analysis showed that Prx4 knockdown led to decreased expression of Twist1/2 and Cyclin D1. Subcutaneous injection of control and Prx4 knockdown DLD-1 cells resulted in smaller tumors in the knockdown group. Prx4-depleted tumors had reduced protein levels of PCNA, N-Cadherin, β-catenin and MMP-9. Finally, the authors found that treatment of DLD-1 with inhibitors of Protein Kinase Cα, RhoA GTPase, ERK1/2 or EGFR increased trimethylation of H3K4 of Prx4 promoter [126]. Thus, this study suggests EGFR-induced Prx4 promotes metastasis of CRC.

A study by Ouyang et al. has suggested that curcumin, a polyphenolic compound with anti-inflammatory properties, protects against late-onset diarrhea side-effect of the chemotherapeutic agent CPT-11 [127]. Curcumin treatment of mice in vivo and IEC-6 cells in vitro reversed the suppression of Prx4 by CPT-11 as detected by western blot analysis. Similarly, treatment of the colorectal cancer cell line LOVO with curcumin alone or in combination with CPT-11 significantly enhanced Prx4 protein expression [128]. In addition, treatment of HT-29 cells with portoamides (which have anti-proliferative activity on certain cancer cell lines) also increased the expression of Prx4 protein [129]. Thus, Prx4 is a promising therapeutic target for CRC prevention or treatment. 

In addition, Prx3 has also been reported to promote CRC. Prx3 mRNA and protein expression correlated with stem cell marker CD133 in an HT29 cell line. Knockdown of Prx3 in CD133 + HT29 cells reduced sphere formation ability and sensitized the cells to 5-fluorouracil-induced cell death. Knockdown of Prx3 also reduced liver metastasis in orthotopic xenograft model [130].

### 4.5. Esophageal Carcinoma and Gastric Cancer

Kobayashi and colleagues have reported higher levels of autoantibodies against Prx4 in the serum of esophageal squamous cell carcinoma and gastric cancer patients compared to healthy donors [131]. Thus, Prx4 antibodies could serve as a potential marker for these cancers. In gastric cancer, it was shown that Prx4 is overexpressed in tissue specimens, and higher expression of Prx4 is associated with shorter survival [132]. Knockdown of Prx4 in AGS and MKN28 cell lines decreased cell proliferation, migration and invasion. The authors also found that Prx4 knockdown decreased the expression of EMT transcription factors Snail and Slug. In esophageal carcinoma, Prx4 has been identified to interact with AGR2, which is one of the proteins highly upregulated in this cancer [133]. AGR2 is an ER chaperone known to promote tumor growth and migration in esophageal carcinoma [134]. Identification of this interaction could provide a new strategy for the development of therapeutics.

### 4.6. Liver Cancer

Prx4 knockout mice had a significantly higher incidence of diethylnitrosamone (DEN)-induced hepatocellular carcinoma (HCC) than wild-type or human Prx4 transgenic mice [135]. After DEN treatment, transgenic mice had lower infiltrated neutrophils, less 8-OHdG positive hepatocytes, lower thiobarbituric acid reactive substances (TBARS) and lower serum levels of aspartate aminotransferase and alanine aminotransferase than WT. Immunohistochemical staining of human HCC tissues revealed that tumors with low Prx4 expression had more hepatic and portal vein invasion, higher 8-OHdG level and were more aggressive [135]. The low Prx4 group also had a significantly reduced overall survival than the high Prx4 group. In vitro, knockdown of Prx4 in PLC/PRF/5 and HepG2 cell lines using siRNAs enhanced ROS levels and decreased cell proliferation. The knockdown-cells also had a higher rate of apoptosis and autophagy. Thus, the study indicates that Prx4 inhibits HCC initiation but may have a dual role in the progression of HCC [135].

A later study reported that Prx4 is oncogenic in HCC. Knockdown of Prx4 significantly reduced both anchorage dependent and anchorage independent colony formation of HCC cells [136]. SMMC-7721 shPrx4 cells resulted in significantly smaller xenograft tumors than shNT cells whereas Prx4 overexpressed cells resulted in significantly bigger tumors than vector control cells. Tail-vein injections in nude mice followed by bioluminescence measurement revealed that shPrx4 cells had significantly lower lung metastasis. HCC cells in suspension culture were found to have lower Prx4 expression than those in an adherent condition [136]. They also had higher cleaved caspase 3 in suspension which is exacerbated by knockdown of Prx4. Conversely, overexpression of Prx4 promoted the survival of HCC cells in suspension. Prx4 overexpression in HCC cells increased expression of total β-catenin protein. Active β-catenin protein also increased, though to a lower extent. However, no change was seen in β-catenin mRNA levels. Subsequent analysis revealed that Prx4 interacts directly with ubiquitin ligase β-TrCP, thus inhibiting ubiquitination of β-catenin. In an anchorage-independent condition, knockdown of β-catenin decreased the growth of Prx4 overexpression cells and increased their susceptibility to anoikis [136]. Similarly, overexpression of β-catenin increased the growth and survival of Prx4 knockdown cells. The authors suggest Prx4 upregulation increases the recruitment of β-catenin to ID2 promoter. ID2 acts downstream of Prx4 to mediate the oncogenic activity. Finally, the long non-coding RNA TP53TG1, which is known to suppress HCC, promotes the ubiquitination and degradation of Prx4 [137]. Thus, targeting Prx4 is a promising therapeutic option for treating HCC. 

Prx2 appears to favor HCC survival. In vitro studies show that knockdown of Prx2 in HCC cell lines reduced expression of cancer stemness markers through increased ROS levels while overexpression of Prx2 promoted stemness [138]. Prx3 also has a dual role in HCC. Depletion of Prx3 in HepG2 cells inhibited cell proliferation through decreased ATP synthesis but promoted invasiveness through degradation of extracellular matrix by downregulation of tissue inhibitors of metalloproteinases-1 (TIMP-1) [139].

### 4.7. Glioma

Prx4 expression is upregulated in human and mouse glioblastoma multiforme (GBM) [140]. Silencing Prx4 expression in vitro in GBM neurospheres reduced cell viability and increased ROS production, DNA damage and apoptosis. Furthermore, Prx4 knockdown decreased radiation resistance in vitro. Combination of Prx4 silencing and irradiation was significantly more effective in killing GBM cells and suppressing colony formation than irradiation alone [140]. In an orthotopic transplantation model, it was found that knockdown of Prx4 increased survival of recipient mice by 35% [140]. The knockdown groups had significantly reduced cell proliferation in infiltrating cells as measured by Ki67 staining and significantly higher DNA damage and apoptosis in tumor sections as measured by P-H2AX and cleaved caspase 3 staining than control groups.

Kim et al. reported in 2014 that Prx4 mRNA is significantly upregulated in mouse high grade-glioma (HGG) cultures [141]. Piperlongumine treatment of HGG cells increased ROS levels and suppressed cell growth, mimicking the effects of Prx4 knockdown. The authors found that after piperlongumine treatment, there was an increase in hyperoxidized form of Prx4 and a corresponding decrease in H_2_O_2_ degradation activity. Furthermore, they also noticed higher levels of ER stress after treatment which could again be attributed to Prx4 inactivation. Knockdown of Prx4 in vitro increased mRNA expression of ER stress and UPR markers as well as ER stress response genes. Analysis of REMBRANDT database revealed that patients with intermediate levels of Prx4 in their gliomas survive significantly longer than those with upregulation of Prx4 [141]. These studies suggest Prx4 is a promising target for treating astrocytoma and glioblastoma.

### 4.8. Melanoma of Skin

Hintsala et al. report an inverse association between age and Prx4 expression in primary tumors [142]. Higher expression of Prx4 in sweat gland cells and cytoplasmic Prx4 expression in endothelial cells were associated with better survival. In an analysis of 111 melanoma patient samples, expression of nuclear Prx1 was found to decrease in different cell types including pigment cells, keratinocytes and endothelial cells compared to benign and dysplastic samples [142]. The same study found that fibroblasts in melanoma patient- derived tissues had lower expression of cytoplasmic Prx1 and nuclear Prx2. This study suggests that Prxs could be used for prognosis and as therapeutic targets for treating melanoma.

### 4.9. Non-Hodgkin Lymphoma and Leukemia

Upregulation of Prx3 and Prx4 transcripts correlates with poor prognosis for diffused large B-cell lymphoma patients [143]. The role of these proteins in initiation of progression of this cancer have not been studied.

Prx4 transcript and protein are downregulated in Acute Promyelocytic Leukemia (APL) relative to AML samples, presumably due to increased levels of H3K27me3 at the transcription start site of Prx4, as indicated by chromatin immunoprecipitation [144]. The molecular mechanism of how the decrease in Prx4 expression might contribute to APL is not understood. In addition, Prx1 is found to be upregulated in activated natural killer cells in vitro [145]. Further studies are warranted to elucidate the function of Prxs in NK cell function.

### 4.10. Oral Squamous Cell Carcinoma (OSCC)

Prx4 was one of the four antioxidants found to be upregulated in tumor samples of OSCC patients compared to adjacent normal tissue [146]. The authors suggest these genes are potential candidates for biomarkers. In addition, Prx1 was also upregulated in mouse xenograft tumors of OSCC cell line SCC15 [147]. Additional studies are needed to understand the functions of Prx1 and Prx4 in OSCC.

### 4.11. Pancreatic Cancer

High expression of Prx4 is associated with liver metastases and lower survival of pancreatic cancer patients [148]. Orthotopic implantation of human cancer cell lines in mice pancreases confirmed that loss of Prx4 increased disease-free survival. Treatment of pancreatic cancer cell lines with 6-aminonicotamide to reduce NADPH levels reversed the decrease in cell proliferation seen upon Prx4 depletion. Therefore, targeting Prx4 has the potential of being beneficial to pancreatic cancer patients.

## 5. Conclusions

**Figure 6:**
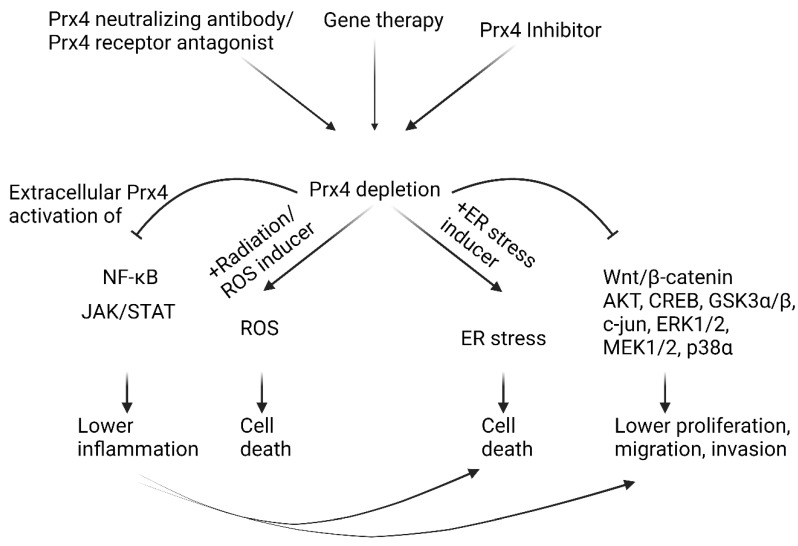
Depletion of Prx4 as a therapeutic approach for cancer treatment.

Redox imbalance in cells affects the integrity of macromolecules and cellular structure and function. Prx4 is an important enzyme that uses its cysteine thiols to protect cells against oxidative stress and ER stress. Chronic stress conditions induce inflammation, which in turn predisposes patients to cancer. Our literature survey confirms that Prx4 tends to suppress inflammation in pathogenesis of inflammatory diseases. However, upregulation of Prx4 is associated with tumor promoting inflammation in highly common cancers including lung and colorectal cancers (see Figure 5). Further studies need to be conducted to understand the role of Prx4 in inhibiting tumor-extrinsic inflammatory triggers while promoting tumor-associated inflammation. In addition, the mechanism of upregulation of Prx4 in different inflammatory diseases and cancer has not been studied. Also not understood are the mechanisms of secretion and functions of secreted Prx4. Finally, identification of specific inhibitors of Prx4 would move the field further forward (Figure 6). Although inhibitors of Prx1 and Prx2, such as ConoidinA and Frenolicin B, are being evaluated for cancer treatment, specific inhibitors against Prx4 are yet to be discovered [149,150]. Thus, Prx4 is a highly promising therapeutic target for cancer prevention and treatment.

## Figures and Tables

**Table 1:** Summary of human Prx4 homologues and conservation relative to human Prx4 generated using Homologene.

Species	Gene Symbol	% Sequence Similarity	
		Protein	DNA
*H. Sapiens*	PRDX4		
*M. mulatta (Rhesus macaque)*	PRDX4	98.5	98.4
*C. lupus (Wolf)*	PRDX4	93	89.2
*B. taurus (Cattle)*	PRDX4	93.8	90.8
*M. musculus (House mouse)*	Prdx4	95	89.1
*R. norvegicus (Brown rat)*	Prdx4	94.5	90.3
*G. gallus (Red junglefowl)*	PRDX4	91.9	81.6
*X. tropicalis (Western clawed frog)*	prdx4	93.6	81.1
*D. rerio (Zebrafish)*	prdx4	88.7	74.8
*D. melanogaster (Common fruit fly)*	Jafrac2	71	64.4

## Data Availability

Not applicable.

## Abbreviations

AD	alzheimer’s disease
APL	acute promyelocytic leukemia
APP	amyloid precursor protein
COX-2	Cyclooxygenase-2
Cp	peroxidatic cysteine
Cr	resolving cysteine
CRC	colorectal cancer
Cys-SOH	sulfenic acid
DEN	diethylnitrosamone
DM	diabetes mellitus
DSS	dextran sulfate sodium
ELISA	enzyme-linked immunosorbent assay
ER	endoplasmic reticulum
Ero1	ER oxidoreductin 1
ERp44	endoplasmic reticulum protein 44
FAD	flavin adenine dinucleotide
GBM	glioblastoma multiforme
GPx	glutathione peroxidase
GSH	glutathione
H_2_O_2_	hydrogen peroxide
HCC	hepatocellular carcinoma
HGG	high grade-glioma
JAK	janus kinase
LUAD	lung adenocarcinoma
MDP	microbial product muramyl dipeptide
MSCs	mesenchymal stromal cells
NAC	N-acetyl cysteine
NAFLD	nonalcoholic fatty liver disease
NF-κB	nuclear factor kappa b
NSAIDs	non-steroidal anti-inflammatory drugs
NSCLC	non-small cell lung cancer
OGD	oxygen glucose deprivation
OSCC	oral Squamous Cell Carcinoma
PDI	protein disulfide isomerase
Prx	peroxiredoxin
QSOX1	quiescin sulfhydryl oxidase 1
RA	rheumatoid arthritis
ROS	reactive oxygen species
SCC	squamous cell carcinoma
SNPs	single-nucleotide polymorphisms
SOD	superoxide dismutase
Srx	sulfiredoxin
STAT	signal transducer and activator of transcription
TBARS	thiobarbituric acid reactive substances
Trx	thioredoxin
